# iPSC Therapy for Myocardial Infarction in Large Animal Models: Land of Hope and Dreams

**DOI:** 10.3390/biomedicines9121836

**Published:** 2021-12-05

**Authors:** Daina Martínez-Falguera, Oriol Iborra-Egea, Carolina Gálvez-Montón

**Affiliations:** 1 Faculty of Medicine, University of Barcelona (UB), 08036 Barcelona, Spain; mdmartinez@igtp.cat; 2 ICREC Research Program, Germans Trias i Pujol Health Research Institute, Can Ruti Campus, 08916 Badalona, Spain; oiborra@igtp.cat; 3 Heart Institute (iCor), Germans Trias i Pujol University Hospital, 08916 Badalona, Spain; 4 CIBERCV, Instituto de Salud Carlos III, 28029 Madrid, Spain; 5 Institut d’Investigació Biomèdica de Bellvitge-IDIBELL, L’Hospitalet de Llobregat, 08908 Barcelona, Spain

**Keywords:** induced pluripotent stem cells, cardiovascular disease, myocardial infarction, large animal models, cardiac regeneration

## Abstract

Myocardial infarction is the main driver of heart failure due to ischemia and subsequent cell death, and cell-based strategies have emerged as promising therapeutic methods to replace dead tissue in cardiovascular diseases. Research in this field has been dramatically advanced by the development of laboratory-induced pluripotent stem cells (iPSCs) that harbor the capability to become any cell type. Like other experimental strategies, stem cell therapy must meet multiple requirements before reaching the clinical trial phase, and in vivo models are indispensable for ensuring the safety of such novel therapies. Specifically, translational studies in large animal models are necessary to fully evaluate the therapeutic potential of this approach; to empirically determine the optimal combination of cell types, supplementary factors, and delivery methods to maximize efficacy; and to stringently assess safety. In the present review, we summarize the main strategies employed to generate iPSCs and differentiate them into cardiomyocytes in large animal species; the most critical differences between using small versus large animal models for cardiovascular studies; and the strategies that have been pursued regarding implanted cells’ stage of differentiation, origin, and technical application.

## 1. Introduction

Heart failure (HF) is the end-stage clinical syndrome for a variety of cardiovascular diseases (CVD) and is currently the leading cause of morbi-mortality worldwide [1]. HF most commonly develops after acute myocardial infarction (MI), when the injured myocardial tissue fails to recover or regenerate [2]. A significant proportion of patients develops pathological ventricular remodeling and progressive HF despite the use of evidence-based medical therapies [3,4]. Although cell-based strategies have emerged for myocardial regeneration after MI, it remains unclear which cell type is optimal for completely restoring cardiac tissue. From the arduous experimental race to find the ideal cell source, has emerged the ability to induce lineage dedifferentiation of easily accessible somatic cells into induced pluripotent stem cells (iPSCs). This has radically opened a promising therapeutic alternative for CVD, since iPSCs are capable of generating an unlimited range and quantity of clinically relevant cell types, including cardiac cell populations [5,6,7].

Like other experimental strategies, stem cell therapy must meet multiple requirements before reaching the clinical trial phase, and in vivo models are indispensable for ensuring the safety of such novel therapies. Most cardiovascular pre-clinical studies using iPSCs have been conducted in small animal models, the findings support their potential therapeutic benefits to improve cardiac function [8,9,10,11]. However, there remains a need for MI translational studies in large animal models to fully evaluate the therapeutic potential of this approach, and to empirically determine the optimal combination of cell types, supplementary factors, and delivery methods to maximize efficacy and stringently assess safety. In cardiovascular research, such studies are usually performed in swine models, but can also use sheep, dog, and macaque models [12], with each species being better suited for specific applications. In this review, we will describe both in vitro and in vivo state-of-the-art techniques for iPSC cardiac differentiation, as well as the main translational studies investigating iPSC cardiac therapy.

## 2. iPSC Generation and Cardiac Differentiation

### 2.1. Obtaining iPSCs from Large Animals

Pluripotent stem cells are defined as being able to differentiate into any cell type, regardless of their germ layer, and as being germline transmitted. Such cells could originally be obtained only by isolating embryonic stem cells (ESCs) from the inner cell mass of early-stage embryos [13,14,15]. However, in 2006, Takahashi and Yamanaka reported that the experimental induction and co-expression of Oct3/4, Klf4, Sox2, and c-Myc could force the retrograde de-differentiation of adult somatic cells into a pluripotent state [16]. To date, murine ESCs are the only cell lines proven to be germline transmitted—the most stringent criteria of pluripotency—and are thus considered true (“naïve”) pluripotent ESCs. Most other ESC/iPSC lines, including those generated from human and large animal cells, share more common characteristics with mouse epiblast-derived stem cells, which cannot form chimeras after blastocyst injection, and are not considered as flexible (“primed”) [17,18,19,20,21]. Indeed, new studies suggest that the molecular mechanisms of the naive state of pluripotency in pre-implantation embryos of other species (including rabbit or primate) may differ from those identified in rodent embryos. Specifically, not all the transcription factors that define naive pluripotency in mice are expressed in their epiblasts [22,23,24], and the signaling pathways that activate these genes and the timing of their upregulation during pre-implantation development also differ [25,26].

**Figure 1:**
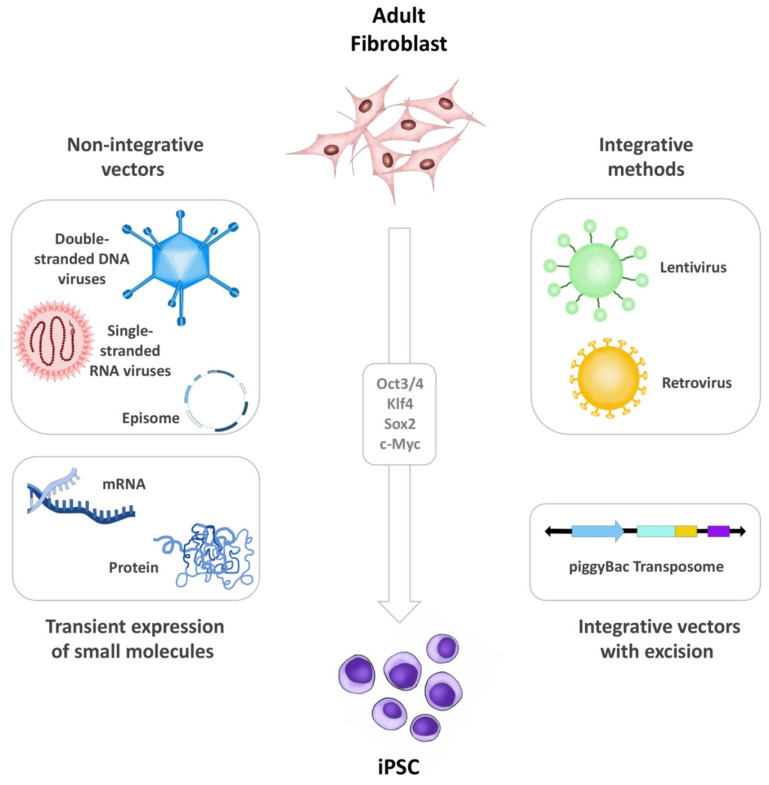
iPSC generation. Illustration summarizing the different methods for iPSC generation including non-integrative vectors, integrative methods, transient expression of small molecules, and integrative vectors that exhibit subsequent excision. The figure was designed and hand-drawn by CG-M.

The first iPSCs were generated through virus-mediated (mainly lentiviruses and retroviruses) transduction of transcription factors, leading to direct integration within the host cell genome, which remains the gold standard of iPSC generation today. However, methods that circumvent genome modification, also called non-integrating techniques, are being extensively developed and evaluated [27,28,29]. Indeed, the dominant trends in reprogramming technology are designed to prevent insertional mutagenesis (by using non-integrative approaches) and contamination of donor cells with co-cultured feeder cells (by using xeno-free conditions and defined extracellular matrix components). The most widely adopted methodologies include the use of direct non-integrative vectors (e.g., single-stranded RNA viruses; double-stranded DNA Viruses and episomes) [30,31,32,33]. Here, Sendai virus (SV), a single stranded negative-sense RNA virus that replicates in the cytoplasm, is widely used in a broad range of research experiments mainly due to its high transduction efficiency, its rapid detectable transgene expression and its auto-erasable nature as a vector [34,35,36]. SV replicates independent of cell cycle, unlike other approaches where the exogenous genes are expressed only as the cell divides, producing very high copy numbers of the target gene. Notably, SV is particularly useful when reprogramming derived blood cells, such as CD34 positive cells, T-cells, and PBMCs, but also work well with fibroblasts, keratinocytes, and other cell-types [37,38]. Other non-integrative methods include integrating vectors that exhibit subsequent excision (e.g., piggyBac transposons) [39,40,41], or transient expression of small molecules (e.g., mRNA or proteins) (Figure 1) [42,43,44,45].

Diverse combinations of vectors and targeted reprogramming genes have been used to generate iPSCs from most farm animals and agriculturally important ungulates [46,47], and these studies highlight some rather interesting commonalities among protocols. Skin fibroblast isolation through biopsy seems to be the preferred cell source for starting iPSC generation, and it appears to be important that cells are obtained from animals that are as young as possible (days old instead of weeks or months). This already indicates a low robustness of the re-programming protocols, as researchers must utilize less mature cells, possibly through epigenetic marks. For instance, many of these iPSC lines exhibit inconsistent expression of typical human and murine pluripotency surface markers, such as SSEA-1, SSEA-3, SSEA-4, TRA-1-81, and TRA-1-60. Moreover, some of these markers display confounding effects, as they can also be expressed in the trophectoderm or in more differentiated stages [48]. The differences in epiblast development among these species could be thwarting reprogramming efforts, leading scientists to generate cells towards different developmental points. This could explain why iPSC generation using large animal models may require different culture conditions and yield varying marker profiles.

The swine model is emerging as the large animal of choice [49]. Literature mining in Pubmed using the search terms: ((“induced pluripotent stem cells”[MeSH Terms] OR (“induced”[All Fields] AND “pluripotent”[All Fields] AND “stem”[All Fields] AND “cells”[All Fields]) OR “induced pluripotent stem cells”[All Fields] OR “ipsc”[All Fields]) AND (“swine”[MeSH Terms] OR “swine”[All Fields] OR “swines”[All Fields])) AND ((journalarticle[Filter]) AND (english[Filter]) AND (2006:2021[pdat])), including only original articles in English language from January 2006 until October 2021, revealed over 300 studies using iPSCs and swine models, of which 103 describe the generation, maintenance, or differentiation of porcine-derived iPSCs (piPSCs), reflecting the clear clinical transfer intention [50,51,52,53,54,55,56,57,58,59,60,61,62,63,64,65]. In contrast, there were only 18 original articles involving iPSCs derived from horse [60,66,67,68,69,70,71,72,73], 15 from cattle [74,75,76,77,78,79,80], 15 from dog [81,82,83,84,85,86], and 14 from sheep/goat [87,88,89,90,91,92,93,94]. In most of these iPSC lines, pluripotency heavily depends on fibroblast growth factor and Activin/Nodal signaling (NANOG and LIN28) [80,95]. In some cases, these factors can replace KLF4 and c-Myc, which have reportedly carried oncogenic potential. Such “primed” colonies are characterized by flattened morphology and can be difficult to passage as single cells. However, by applying selective growth procedures immediately following reprogramming, it is possible to generate LIF/STAT3-dependent iPSCs (which more closely resemble naïve status) from swine cells [96].

Several reports show that additional strategies to enhance pluripotency yield and capabilities can drastically improve iPSC generation and maintenance. Wu et al. recently reported that m6A modification (the most prevalent modification in eukaryotic mRNAs) modulates the SOCS3/JAK2/STAT3 pathway, and thereby plays an important role in regulating the pluripotency of porcine-derived iPSCs [97]. Therefore, de-methylation protocols (such as those employing 5′-AZA-2′deoxycytidine) performed prior to de-differentiation efforts can have significant impacts on the success of iPSC generation [98]. Other strategies include the use of iPSC lines established from murine origin to extract nuclear and cytoplasmic factors that can be later transfected into host cells. In such methods, researchers aim to blindly add varying cellular contents that, although largely unknown, could hypothetically play a role in reprogramming, and thus could improve the de-differentiation process (a form of shotgun approach). Notably, the transcriptomic profiling of pluripotent cells highlights that pluripotency and cell expansion could be increased by the addition of specific small molecules to culture media, or the use of more refined media [99]. Bingbo et al. recently described that IRF-1 expression in the inner cell mass of a porcine early blastocyst enhances the pluripotency of piPSCs, partly through promotion of the JAK-STAT pathway [100]. The authors performed ChIP-Seq analysis, which revealed that IRF-1 activates genes related to the JAK-STAT pathway, and expression of IL7 and STAT3. Inhibition of STAT3 phosphorylation reverted the expression of primed genes in IRF-1-overexpressing cells, while addition of IL7 in culture medium resulted in no apparent changes in the cell morphology, anatomopathological staining results, or expression of pluripotency-related genes. Additionally, IRF-1 knockdown during reprogramming appeared to reduce reprogramming efficiency, whereas IRF-1 overexpression yielded the opposite effect. Such studies are uncovering a plethora of transcription factors that play key roles in optimizing pluripotency.

Most importantly, these studies show that species-specific, human, and mouse transcription factors, as well as combinations of transcription factors from different species, can be used to reprogram other animal cells.

### 2.2. Cardiac Lineage iPSC Differentiation in Large Animal Models

Direct iPSC delivery through transplantation or injection has become the most studied approach in regenerative medicine [101,102,103,104,105,106,107]. However, iPSC translational efforts have been dampened by concerns regarding the teratogenic potential of undifferentiated cells, as well as the lack of timely stimuli to carefully guide the in situ differentiation of these cells [108]. Therefore, increasing research attention has been focused on the in vitro differentiation of iPSCs in the laboratory setting, to obtain the desired cell type, which can then be applied to the patient. Notably, the differentiation of murine and human iPSCs into cardiomyocytes (iPSC-CMs) has been extensively described and is now standard practice in laboratories worldwide [109,110,111,112,113,114,115,116,117,118,119].

**Figure 2:**
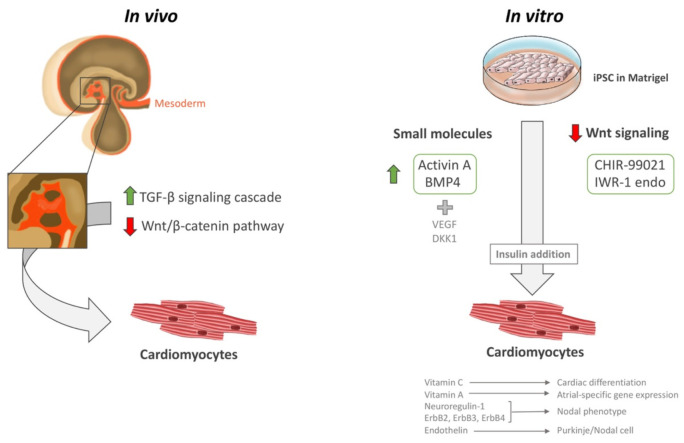
iPSC cardiac differentiation. Illustration showing the in vivo and in vitro methods for iPSC cardiac differentiation, including small molecules and Wnt signaling downregulation. Insulin is a necessary step to mature CMs into a beating state. The figure was designed and hand-drawn by CG-M.

In vivo, it is thought that cardiac progenitor cells initially originate from the mesoderm following activation of the tumor growth factor-β (TGF-β) signaling cascades, and subsequent inhibition of the Wnt/β-catenin pathway (Figure 2). Most cardiac differentiation protocols attempt to replicate distinct stages of this developmental process and fall into two categories: small molecule-based or based on WNT signaling pathway inhibition. Briefly, both protocols usually involve growing purified iPSC monolayers in Matrigel (Corning Life Sciences, Glendale, CA, USA) up to 90% confluence before starting the reprogramming procedures. Next, the small molecule-based protocols usually entail the activation of Activin A and BMP4 (and sometimes the use of specific cytokines, such as VEGF and DKK1) in a timed sequence. On the other hand, WNT signaling pathway inhibition involves the use of CHIR-99021 (2 mg) and IWR-1 endo (10 mg) (Figure 2) [120,121,122,123,124,125,126,127,128]. Moreover, insulin is required to observe functional beating, but is counter-productive during initial stages, as it inhibits differentiation. Thus, almost all protocols start with insulin-free media, and involve insulin addition only after reprogramming has been established [129,130,131,132,133,134].

Certain vitamins and cytokines also reportedly help in cardiac differentiation and influence the efficiency and specificity of the resulting cells. For instance, ascorbic acid (vitamin C) promotes cardiac differentiation of pluripotent cells, and retinoic acid (vitamin A) promotes atrial-specific gene expression [129,135,136,137,138]. A signaling pathway involving neuroregulin-1 (NRG-1; an important regulator of cardiac development and function) and the receptor tyrosine kinases ErbB2, ErbB3, and ErbB4 orientates cells towards a nodal phenotype [139]. Additionally, endothelin, a paracrine factor secreted by endothelial cells in the arterial walls, is apparently involved in Purkinje/nodal cell differentiation [140].

While some studies show that these cells can efficiently couple to the injured myocardium and restore cardiac function, many have also reported arrhythmogenic issues. To explain this, several studies demonstrate that iPSC-CM cultures can be highly heterogeneous, comprising both ventricular- and nodal-like CMs. Nonetheless, prominent works have demonstrated regenerative capacities when non-human primates are transplanted with allogenic iPSC-CMs in a post-MI setting. In 2016, Shiba and colleagues reported that injected iPSC-CMs electrically coupled with host CMs resulted in improved cardiac contractile function at 4 and 12 weeks after transplantation [114]. However, these cells clearly elicited transient episodes of ventricular tachycardia, and the study only included five subjects transplanted with a single iPSC line. Moreover, the authors noted that the 12-week observation period after cell transplantation does not allow a definitive conclusion regarding graft survival without chronic rejection.

Unfortunately, few studies have examined the use of autologous or allogeneic iPSC-CMs in large animal models. As the end-goal in biomedicine is the clinical applicability of such therapies, human iPSC-CMs are the cell type of choice in the vast majority of investigations. Together with the ease of obtaining iPSC lines of human origin compared to from other large animal models, this has led to predominant use of human iPSCs to test stem cell therapy in other species [141,142]. In such experiments, animals must be immunosuppressed to maintain the cells after transplant. Although such practices are faster and easier to implement, the result cannot be considered to faithfully reproduce most of the diseases under investigation. Most cardiac differentiation protocols currently employed in large animal models are directly extrapolated from those effective in human and murine cells. However, as the murine protocols do not work well with human cells and vice versa, there is little scientific basis to think that either of these would optimally translate to other animal species (with the exception of human protocols that reportedly work well in other primates). Yet, few efforts have been directed towards developing better more specific protocols for use in other large animals, which impedes the development of more reliable pre-clinical models.

## 3. Large Animal Models for Translational Cardiovascular Studies

### 3.1. The Importance of Large Animal Models in Cardiac Stem Cell Therapies

When approaching the development of new treatment, to justify the huge time and economic efforts required, it is critical to carefully select what animal models should be used to determine the likelihood that a therapeutic approach will be clinically successful [143,144,145,146]. Although no animal model can ever completely substitute for human studies, large animal models have supplied critically important information about how humans might respond to specific therapeutic strategies. Most importantly, when an intervention improves the main outcomes in a well-designed trial using any experimental model, this proof of concept is a valuable piece of evidence guiding further studies. Compared to data from small species, well-designed large animal studies can better predict human trial outcomes [147,148,149].

### 3.2. Critical Differences between Large and Small Animal Models in Pre-Clinical Studies

From the practical standpoint, small animal species have tremendous advantages for pre-clinical research [150,151,152]. The high availability of reagents to test molecular pathways, and the relatively low cost of studying a large number of subjects, facilitate the derivation of mechanistic insights relating to the therapeutic being studied. Specifically, mice have been a species of choice for studies of stem cell biology in mammals, due to their reduced cost, high offspring generation, and ease of genetic manipulation.

Nevertheless, positive results in small animal models (mostly murine) have frequently been followed by clinical trials failing to confirm efficacy, justifying skepticism regarding the reliability of findings obtained in studies of small species [153,154,155]. These negative outcomes may be explained by the lack of disease complexity in the animal model used; and by the differences between rodents and humans in terms of size, life span, heart rate, and the innate and adaptive immune response systems. Moreover, different pathologies that can be more easily induced in small models (such as hypertension, diabetes mellitus, or hypoxia) are used as proxy to imitate some of the cellular and physiological changes in human diseases, which limits the capacity to extrapolate findings in the animal models. Many of these models fail to precisely recapitulate particular human disease phenotypes, especially in stem cell research, which has compelled investigators to examine animal species that may be more predictive of humans.

Compared to mice, large animals are often better models of human disease phenotypes [156,157,158,159,160], due their physiological factors are close to humans. Moreover, large animals also have similarities in terms of number and types of stem cells that can be reproducibly extracted and handled in sufficient amount for analysis and for different applications. Key drivers of translational applicability include advances in experimental surgery, and the ability to use equipment and techniques developed for human applications for cell delivery and animal monitoring. These features let researchers to study the safety of applications, dosages of biologics, and a delivery method that can be easily translated to humans. This is the case of the development of different approaches and techniques, such as surgical options and imaging technologies. Large animal models allow to test survival, activation and differentiation of the implanted cell by non-invasive monitoring. Clearly, it is undesirable to interpret the results of highly interventional therapies by performing experiments solely in small animal models and extrapolating the conclusions to human trials [161,162].

This is not to imply that large animal models do not also have intrinsic limitations. The most important limitation is the high cost of animal maintenance. Research in large animal species involve larger and specialized housing and surgical facilities, including higher costs associated to feed, veterinary care, and surgical costs. Additionally, their longer reproductive cycles and slow growth rates make that pre-clinical trials are slower and less economical. More specifically relevant to cell therapy, research in large animals is complicated by the relative absence of stable and well-characterized stem cell lines and protocols for their maintenance, differentiation, and cell status monitoring; and the limited availability of species-specific antibodies, expression microarrays, and other research reagents. Finally, complex diseases are difficult to fully capture with any model and when considering all previous points, studies involving large animal models can end up being poorly designed and insufficiently powered. In these studies, costs have a big impact in the design, which many times constrain the budget and forces to include too few animals in each study arm, so that false-positive outcomes can often occur by chance alone.

Nevertheless, since large animal models provide a setting that is closer to the human situation than those found in rodent models, large species studies are essential to justify the risks and costs of clinical trials [147].

### 3.3. Pre-Clinical Stem Cell Research for Cardiovascular Diseases

Among larger animal species, dogs, pigs, sheep, and non-human primates are suitable models for CVD-related studies. These animals have physiological parameters similar to humans (Table 1), and their size allows the use of echocardiography and cardiac magnetic resonance imaging techniques that are already widely used in the clinic, which yields information that is more rapidly transferrable and relevant.

These large species have exhibited marked improvement in cardiac function following stem cell treatments using a variety of cells, including skeletal myoblasts, bone marrow and adipose tissue-derived stem cells, cardiac stem cells, and endothelial adult stem cells [163,164,165]. Moreover, meta-analysis studies involving large animals that have received cardiac stem cells as therapy for ischemic heart disease clearly demonstrate that these models can predict clinical trial outcomes, and that such treatments are safe; however, the specific reported cardiac improvements are mixed and the gathered datasets very heterogeneous [149]. These studies can potentially address a variety of important issues before the performance of clinical trials, including determination of the optimal cell type and delivery method, time of administration, and types of clinical condition for which a treatment can be beneficial.

## 4. Applications of iPSC-Based Therapies in Large Animal Models of MI

All the iPSC-based therapies in large animal models are summarized in Table 2. Key published literature was searched using PubMed. We applied the search terms: (“induced pluripotent stem cells” OR “ipsc”) AND (“large animals” OR “swine” OR “pig”) and (“induced pluripotent stem cells” OR “ipsc”) AND (“large animals” OR “swine” OR “pig” AND (“myocardial infarction”) including only original articles in English language until October 2021.

### 4.1. Undifferentiated iPSCs

Some groups have generated piPSCs, opening up a wide range of possibilities to conduct pre-clinical testing in pigs without requiring immunosuppressive treatment [48,55,56,181]. However, good differentiation of piPSCs to CMs has not yet been achieved, and most piPSC studies performed in swine have used undifferentiated cells. Nevertheless, one major concern is the possibility of uncontrolled tumorigenesis after stem cell administration [182] and studying allogeneic transplantation in immune-competent pigs could reveal whether the immune system can control undifferentiated cells that may remain after transplantation.

In 2013, Li and colleagues published the first study assessing intramyocardial injection of allogeneic piPSCs in a pig model of MI [166,167]. First, they tested intracoronary administration of piPSCs, and found no effect on the infarct zone or in terms of cardiac perfusion, mainly due to cell washout through the blood circulation. Thus, to ensure piPSC engraftment, they performed a second study testing the direct intramyocardial injection of 2 × 10^7^ piPSCs into infarct zones (8 sites) and border zones (12 sites). Six weeks after injection, treated animals exhibited an improved left ventricular ejection fraction (LVEF), better myocardial perfusion (likely due to vascular endothelial cell differentiation of the engrafted iPSCs), less oxidative stress, angiogenesis in the border zone, upregulated connexin 43 expression, and less ventricular tachycardia inducibility. Importantly, no tumors were detected in any animal, despite the use of undifferentiated cells. Collectively, their data demonstrated that direct intramyocardial injection of piPSCs is safe and can decrease infarct size and improve cardiac function; however, the slight reported improvements were clinically irrelevant.

In 2014, the same research team published another study using an identical piPSC cell line, dose, and delivery route in an immune-suppressed MI porcine model to alleviate the non-specific immune response and the acute inflammatory reactions [168]. At 6 weeks after treatment, the iPSC group showed less pronounced LV structural abnormality and cardiac dysfunction and accompanied again by a slightly reduced scar size. Notably, most of the transplanted cells were found in the border zone, and they had differentiated into vascular endothelial cells (ECs), which were integrated into pre-existing vessels or generating new vessels, and to a lesser extent into myoblasts. Thus, iPSC transplantation was associated with significantly increased vascular density and reduced myocardial apoptosis in the border zone. Moreover, the iPSC group exhibited significantly increased proangiogenic and antiapoptotic factors, and significantly attenuated CM hypertrophy. In conclusion, these results suggested that piPSC transplantation can result in cardiac functional recovery, mainly by promoting angiogenesis, inhibiting apoptosis, and ameliorating cardiac remodeling.

Years later, our group conducted another study that proved the safety of transplanting allogeneic piPSCs using three different engineered constructs that were implanted into an immune-competent MI swine model [169]. We tested the adipose graft transposition procedure (AGTP) [183], an acellular human pericardial scaffold (scaffold), and a combination of both (AGTP-scaffold), with or without 0.5–1 × 10^6^ piPSCs, which was a lower cell concentration than used in the previously described works. Thirty days after implantation, histopathological analyses confirmed no presence of piPSCs within the host myocardium or biomatrices. The AGTP-scaffold group showed significantly higher vascularization, irrespective of piPSC delivery, in both the infarct and border zones. Consistent with the disappearance of the implanted cells, and unlike in other studies, these treatments did not yield functional benefit in terms of LVEF, cardiac output, ventricular volumes, or necrotic mass. On the other hand, histopathological examination of the heart at 90 days of follow-up confirmed the absence of teratoma formation in all animals. Therefore, we concluded that residual undifferentiated piPSCs should pose no safety concern when used in an allogeneic context in immune-competent recipients, at least in cardiac regenerative medicine.

Taken together, the above-mentioned studies have demonstrated that allogeneic iPSC therapy is safe in terms of teratogenesis; however, no clinical benefits are obtained due to the host immune reaction against the delivered cells.

### 4.2. Differentiated iPSCs

Theoretically, iPSCs have the capacity to differentiate into any other cell type present in the body, independent of the germ layer of choice. This process can be directed by using specific gene regulation methods that target highly conserved developmental molecular pathways, with each differentiation protocol being specific to the desired cell line. Undifferentiated iPSCs are more flexible at delivery but are much less effective at improving tissue-specific problems. In contrast, iPSC-derived cell lines have the potential to more effectively target distinct cellular dysfunctions and have been the central focus of iPSC-based therapeutic research for some years now.

#### 4.2.1. Allogeneic iPSC-CMs: Non-Primate Models

Autologous iPSC transplantation therapy avoids the need for immunosuppression and related problems, such as malignancy and infection. However, the clinical application of this approach is limited by high costs, safety concerns, and challenges related to manufacturing and regulation. To overcome these limitations, an iPSC bank has been developed to store iPSC lines with established safety, with the aim of transplanting iPSC derivatives in an allogeneic manner. However, as previously stated, a potential disadvantage of allogeneic transplantation lies in the immune reaction response, which is responsible for graft rejection [184]. The major histocompatibility complex (MHC) plays an essential role in the post-transplant immune response [185,186]. Therefore, donor/recipient MHC matching can decrease the rejection rate following organ and cell transplantation [187]. To that end, the establishment of iPSC lines from healthy donors with homozygous MHC alleles could be useful for minimizing the number of banked iPSC lines [188,189].

To that end, Kawamura and co-workers generated iPSC-CMs with a homozygous MHC HT1 haplotype line from the cynomolgus macaque, which has an MHC structure identical to that of humans [170]. They transplanted 3.3 × 10^6^ iPSC-CM sheets at the subcutaneous level and by intramyocardial injection in MHC-matched macaques (with heterozygous MHC haplotypes) and MCH-mismatched macaques (without identical MHC alleles) in conjunction with immune suppression treatment. Compared to the MHC-mismatched group, the MHC-matched group displayed a higher engraftment rate and less infiltration of immune cells (CD3^+^ and CD4^+^ T cells). However, MHC-matched transplantation with single or no immune-suppressive drugs still induced a substantial host immune response to the graft. Thus, although MHC-matched transplantation reduced the immunogenicity of allogeneic iPSC-CMs, successful engraftment still required appropriate immune suppression.

Additionally, in 2016, another study examined allogeneic iPSC intramyocardial transplantation in a non-human primate model [114]. Two weeks after MI, 4 × 10^8^ iPSC-CMs were injected into the infarct and border zones of MHC-matched and MHC-mismatched monkeys under immunosuppressive treatment. In the MHC-mismatched group, implanted iPSC-CMs were thoroughly rejected due to severe infiltration of T lymphocytes at 4 weeks after transplantation. However, in the MHC-matched group, the grafted iPSC-CMs survived for 12 weeks with no evidence of immune rejection and exhibited electrical coupling with host CMs. Despite evidence of improved cardiac contractile function at 4 and 12 weeks after iPSC-CM transplantation, the incidence of ventricular tachycardia was significantly increased compared to vehicle-treated controls. No animal showed tumor formation. Collectively, their data demonstrated that allogeneic iPSC-CM transplantation regenerates the infarcted non-human primate heart. However, there remains a need for further research on controlling post-transplant arrhythmias.

Most recently, Sawa’s group tested the effects of iPSC-derived cardiac sheet transplantation in both MHC-matched and MHC-mismatched immunosuppressed macaques. In line with previous findings, the treated animals showed significantly improved cardiac function with less fibrosis and higher vascular density, compared to sham animals, and homozygous MHC haplotypes were preferred to avoid immune rejection. Unfortunately, no arrhythmic inducibility analysis was reported [171].

#### 4.2.2. Xenogeneic hiPSC-CMs

Recent studies have reported methods for the highly efficient differentiation of CMs from hiPSCs, which show typical electrophysiological function and pharmacological responsiveness [190,191]. Transplantation of hiPSC-CMs would mechanically contribute to directly improving cardiac function, among other benefits. However, low retention of the transplanted cells remains a primary factor limiting the effectiveness of this cell therapy [173,192,193,194].

In the onerous fight to identify the ideal approach to ensure hiPSC-CM engraftment, three different techniques have been tested: cell sheets, intracoronary infusion, and intramyocardial injections. Direct intramyocardial or intracoronary injections of dissociated single cells yields an engraftment rate of <10% immediately after transplantation [195,196]. Therefore, some studies have focused on developing innovative new injection techniques to improve cellular retention, such as co-transplantation with human MSCs that release antiapoptotic factors [197]. Moreover, in recent years, various new tissue engineering approaches, including cell sheets, have been developed to enhance cell delivery in myocardial regeneration therapy. In contrast to the needle injection technique, a cell sheet can drive a large number of cells to damaged tissue without transplanted cell loss or injury to the host myocardium. Furthermore, the arrangement of hiPSC-CMs in three-dimensional patches promotes their continuous maturation [174,198,199].

Dr. Sawa’s lab has acquired expertise in generating hiPSC-CM sheets. In their first study in a porcine MI model, cell sheets generated from 2.5 × 10^7^ hiPSC-CMs yielded improved cardiac function (LVEF) and myocardial perfusion, attenuating LV remodeling. Furthermore, treated animals consistently exhibited significantly less accumulation of interstitial fibrosis in border zones, and increased myocardial vascular density, mainly through paracrine effects. The lack of teratoma formation in animals that received hiPSC-CM sheets confirmed the safety of this treatment [176]. However, the authors reported poor engraftment of the transplanted cells, affecting the long-term effectiveness of the treatment. This low cell retention was attributed to ischemia caused by poor vascularization and inflammation in the transplanted sites, as also suggested in other studies [200,201,202]. To overcome this issue, Sawa’s group has focused on a new approach using an omentum flap [177,178]. The omentum is a vascular-rich organ that contains abundant angiogenic factors and has anti-inflammatory effects and was thus expected to help to sustain a blood supply for the cell sheets [203]. The group examined the survival of hiPSC-CMs, enriched with commercial human mesenchymal stem cells (hMSCs), with or without an omentum pedicle, after transplantation into healthy mini-pigs [177] and a mini-pig MI model [178]. They observed significantly improved hiPSC-CM survival in mini-pigs with the omentum than without omentum. Over three months, both groups showed a steady decrease of cell survival, but the proportion of the decrease was significantly less in the omentum group. Moreover, the omentum group exhibited increased capillary density, and upregulated VEGF, SDF-1, and bFGF expression in the transplanted area. Therefore, the omentum pedicle yielded enhanced hiPSC-CM survival in vivo, and produced longer therapeutic effects after MI.

Most recently, studies have tested the addition of different proteins to ensure hiPSC-CM retention and proliferation in swine. Notably, Ye’s lab reported that thymosin β4 (Tb4) improves the engraftment of hiPSC-CM fibrin scaffolds in a porcine model of sub-acute MI [101]. They demonstrated that co-treatment with Tb4 significantly enhanced hiPSC-CM engraftment, induced vasculogenesis, promoted proliferation of CMs and ECs, improved LV systolic function, and reduced infarct size. Moreover, hiPSC-CM engraftment was not correlated with incidence of ventricular arrhythmia, and no tumorigenesis was detected in the immunosuppressed animals. Correspondingly, Zhao et al. have shown that hiPSC-CMs overexpressing cyclin D2 were associated with improved LV function; reduced infarct size; less fibrosis, ventricular hypertrophy, and CM apoptosis; and increased vessel density [179].

Preclinical and clinical studies of stem cell-based cardiac regenerative therapy have widely applied intracoronary infusion and intramyocardial injection (either via direct open-chest or transendocardial catheter-based injections) [204]. Intracoronary cell delivery into a recanalized infarct-related or target artery is safe and practical (e.g., it can be performed using standard balloon catheters); however, its effectiveness is compromised by the low cellular retention in the damaged myocardium. Compared to the intracoronary route, direct intramyocardial injection often results in slightly better retention [205]. Several strategies have been investigated for improving transplanted cell engraftment and viability—including cell aggregation, biomaterials or scaffolds, and pro-survival factors [122,206,207].

Fukuda’s lab has generated spheroids of purified hiPSC-CMs and used gelatin hydrogel as a biomatrix to enhance engraftment [180,206] and developed a new injection device for optimal 3D distribution of these materials in the myocardial layer [208]. They have demonstrated that aggregated CMs (spheroids) are less likely to be cleared from the host heart by normal circulation compared to non-aggregated CMs [206], and that gelatin hydrogel enhances CM engraftment [180]. Their new device comprises six needles, each with six elliptical holes facing in various directions, and with a domed tip to minimize damage to the myocardium. Compared to conventional needle injection procedures, direct epicardial injection of spheroids resulted in better distribution and retention in a layer within the myocardium, and no detrimental effects on cell viability, spheroid shape, or size were detected in healthy pigs. Interestingly, cell injections in gelatin hydrogel increased the retention of beads by approximately 20-fold compared to the retention rate using saline. Furthermore, they verified that their injection device provided a more equal three-dimensional distribution of transplanted cells in all layers of the host myocardium compared to conventional procedures [208].

Most recently, Kawaguchi et al. transplanted intramyocardially hiPSC-derived cardiac spheroids in pigs after cryoinjury. In line with previous works, treated animals exhibited improved cardiac function and reduced infarct size. However, the engraftment of CMs was unusual, and the treated pigs suffered ventricular arrhythmic events correlated with tachycardia [103].

#### 4.2.3. Combination of Multiple hiPSC-Cardiovascular Lineage Cell Populations: Cardiomyocytes, Endothelial, and Smooth Muscle Cells

Both cardiac muscles and vessels are excessively damaged following MI. Therefore, therapeutic strategies should be focused on comprehensively repairing both together, to achieve true cardiac repair. In vitro data strongly suggest that CMs exhibit better survival and resistance to hypoxic injury when co-cultured with ECs and smooth muscle cells (SMCs) than when cultured alone [172,209,210]. Thus, co-administration with ECs and SMCs could enhance the engraftment of transplanted CMs, as well as improve LV myocardial perfusion, cardiac metabolism, and contractile activity through release of signaling molecules [211,212,213]. Consistent with this hypothesis, some groups have tested the differentiation of iPSCs to ECs and SMCs, and their combination with iPSC-CMs and growth factors to treat MI.

In 2014, Ye et al. transplanted hiPSC-CMs, -ECs, and -SMCs into injured hearts of a porcine MI model, with comparison of two delivery methods: through direct intramyocardial injections or through a fibrin patch loaded with insulin growth factor 1 (IGF-1)-microspheres [172]. Four weeks after transplantation, the cell + patch + IGF animals showed the best engraftment rate, ~20-fold greater than achieved with any other delivery method used in the porcine MI model [214]. Their results also demonstrated that co-administration of multiple hiPSC-cardiovascular linage cell transplantation significantly improved LV function, vascular density, and cardiac metabolism; and yielded reductions of infarct size, ventricular wall stress, and apoptosis, without inducing ventricular arrhythmias. The increased angiogenesis was related to paracrine effects, as suggested by other studies [209,211]. Together, these findings reveal that co-administration of trilineage cardiac cell populations, conjugated by microspheres of IGF-1, improves engraftment and graft effectiveness.

Most recently, clinical-sized cardiac tissue sheets (L-CTSs) comprising hiPSC-CMs and hiPSC-derived vascular cells (ECs and vascular mural cells) were also evaluated in a porcine MI model [173]. Transplantation of L-CTSs restored wall motion of the transplanted region, improved cardiac function in terms of higher LV systolic function and LVEF, and yielded reduced fibrosis and greater capillary density in the border region. L-CTS transplantation induced neovascularization, prompting CM hypertrophy attenuation and reducing global LV remodeling, as reported in previous works [215,216]. Once again, the authors stated that the therapeutic mechanism was mainly mediated by paracrine mechanisms, considering that the small engrafted cells cannot be responsible for the reinforcement of mechanical ventricular contraction.

In the same year, Gao et al. developed human cardiac muscle patches (hCMPs) by suspending 4 × 10^6^ iPSC-CMs, 2 × 10^6^ iPSC-SMCs, and 2 × 10^6^ iPSC-ECs in a fibrin scaffold that covers the acute ischemic area in pigs [174]. The cell engraftment rate was 11% at 4 weeks after transplantation, and the hCMP treatment was associated with significantly improved LV function, reduction on infarct size, myocardial wall stress, and myocardial hypertrophy, and less apoptosis in the border area. Notably, the exosomes released from the hCMP exhibited cytoprotective properties that improved CM survival and neovascularization activity, and protected myocytes of the border from apoptosis. These paracrine mechanisms, as well as the microenvironment of the patch itself, likely contributed to the relatively high engraftment rate, which is consistent with the results from studies in rodents [11,217,218].

#### 4.2.4. iPSC-Derived Mesenchymal Stem Cells

Besides iPSCs, mesenchymal stem cells (MSCs)—which can be obtained from several tissues, including bone marrow, fat, placenta, Wharton’s jelly, etc.—have been exhaustively examined as another promising cell type for HF treatment. The differentiation of MSCs from iPSCs (hiPSC-MSCs) has also been successfully tested, and some groups have demonstrated that hiPSC-MSCs exhibit better proliferative capacity, survival, and therapeutic efficacy for tissue repair than bone marrow-derived MSCs [219,220,221]. In parallel, Liao et al. compared the safety and efficacy of human embryonic stem cell-derived CMs (hESC-CMs) vs. hiPSC-MSCs following transplantation in a porcine model of MI-induced HF [175]. At 8 weeks post-transplantation, both cell groups exhibited significantly improved LV function. However, the percentage of infarcted area normalized to body weight did not significantly differ in the hiPSC-MSC group or hESC-CM group compared with in the MI group, suggesting that cell transplantation did not lead to cardiac regeneration. Notably, only the hiPSC-MSC group exhibited markedly increased vessel density and expressions of TGF-α and VEGF-A. These results show that hiPSC-MSCs can stimulate angiogenesis by upregulating the myocardial expression of angiogenic cytokines after transplantation. Moreover, histological assays confirmed the immunomodulatory potential of the hiPSC-MSCs, which activated regulatory T cells and reduced inflammatory cells in the myocardium.

## 5. Conclusions

Preclinical studies in large animal models of MI indicate that iPSC therapy might be a promising approach for treating CVDs. However, cell therapies are notably complex, and studies in large animal models could be performed to fine-tune several factors to further improve the clinical outcomes resulting from such advanced treatments. Specific aspects requiring further investigation include the optimal delivery method (cell sheets vs. intramyocardial injections), and type of cell administered (undifferentiated vs. iPSC-CMs vs. co-administration of multiple iPSC-derived cardiac cell types).

The desirable stem cell for regenerative medicine should be immunologically compatible, easily accessible, with high in vitro expansion, long-term survival, and able to integrate into the host target tissue. Currently, the MSC are the most extensively cell source tested in preclinical and clinical trials, due its easy isolation, high plasticity, and immunomodulation potential [222]. However, results show that the benefits are modest and mainly due to their paracrine effect, not to their nesting and differentiation in the host tissue. Contrary, iPSC, despite their capacity of teratoma formation (that can be evaded by a previous differentiation into committed lineages), are able to migrate, nest and integrate with the host myocardium, acting as new functional cardiac cells. Nevertheless, iPSC therapy not only still requires high immunosuppression to ensure its safety and effectiveness, but also their arrhythmic potential has to be solved before reaching the clinical setting. Considering the results of the preclinical studies with MSC and iPSC, it is difficult to conclude which of the two cell sources offers more notable positive outcomes. In this sense, MSCs, due to their immunomodulatory potential, may offer greater benefit in the context of acute MI when the inflammatory plethora of cytokines is active and on which the extension of the definitive myocardial scar (tissue at risk) depends. On the other hand, iPSC-CM could be a promising therapy not only for acute but also for chronic MI, where cardiac regeneration is feasible. Taken together, one possibility to consider in the search for the best therapeutic results lies in the joint administration of MSC and iPSC to simultaneously resolve post-MI inflammation, iPSC rejection, as well as cardiac regeneration, converging in a cardiac functional improvement.

The available data show that co-administration of hiPSC-cardiovascular lineage cell populations conjugated with certain biological molecules yield the most robust response in terms of promoting myocardial repair, as this method comprehensively approaches all injured tissues of the heart (rather than only CMs) to achieve true cardiac repair. Additionally, cell-sheet delivery systems are probably the most effective for delivering large numbers of cells without cell loss due to post-transplantation physical strain, hypoxia, or cell washout through the vascular or lymphatic system. However, a primary factor limiting the efficacy of this approach is the low proportion of transplanted cells that survive in the recipient heart just a few weeks following graft administration. Many research groups have begun examining this problem and are providing innovative solutions to improve the cell engraftment rate—such as new injection techniques, combination of the cell sheets with the omentum flap, or adding specific proteins. Additional research is clearly still needed to overcome engraftment issues, as well as to study more intricate cellular interactions, such as innervation (critical to the electric coupling of the graft and recipient tissue), vascularization, and final integration, it has been less than 15 years since the discovery of iPSCs, let alone since their application as stem cell therapy. If the progression of future research resembles the success over the past decade—as indeed seems to be the case—there will still be plenty of opportunities to marvel at the solutions that have yet to come and that will render stem cell approaches routine clinical practice.

## Figures and Tables

**Table 1:** Cardiac similarities and differences of large animals compared to humans.

Specie	Similarities	Differences
**Canine**	Closed circulatory system composed by heart, veins, and arteries Physiological function of the heart Heart anatomy (4 chambers) Hemodynamic parameters	Anatomic variation in the thoracic cavity Collateral coronary circulation Smaller heart size Resting heart rate (60–160 bpm) Number of pulmonary veins (4–8) Presence of the left azygous vein Positioning of vena cava Size and shape of the atrial appendage Higher heart weight/body weight ratio Tricuspid valve with 2 leaflets Physiological respiratory arrhythmia
**Swine**	Closed circulatory system composed by heart, veins, and arteries Physiological function of the heart Heart anatomy (4 chambers) Heart size Coronary anatomy Analogous coronary pattern Lack of collateral coronary circulation Left coronary supplies the majority of the myocardium Hemodynamic parameters Heart weight/body weight ratio Valvular anatomy	Anatomic variation in the thoracic cavity Number of pulmonary veins (2) Higher content of Purkinje fibers Higher presence of numerous nerves Presence of the left azygous vein Faster sinus rhythm Shorter PR interval
**Ovine**	Closed circulatory system composed by heart, veins, and arteries Physiological function of the heart Heart anatomy (4 chambers) Lack of collateral coronary circulation Heart rate	Anatomic variation in the thoracic cavity Lower heart weight/body weight ratio Shorter and immobile ascending aorta Structure and composition of the valves Intervalvular or membranous septum absent
**Non-human primates**	Closed circulatory system composed by heart, veins, and arteries Physiological function of the heart Heart anatomy (4 chambers) Lack of collateral coronary circulation Genomic organization of the MHC region Genetic and metabolic parameters Ratio of heart weight to body weight	Faster heart rate

**Table 2:** **iPSC studies in large animals.** MI: myocardial infarction; HF: heart failure; hESC-CMs: human embryonic stem cell-derived cardiomyocytes; hiPSC-CMs: human induced pluripotent stem cell-derived cardiomyocytes; hiPSC-ECs: human induced pluripotent stem cell-derived endothelial cells; hiPSC-MSCs: human induced pluripotent stem cell-derived mesenchymal stem cells; hMSCs: human mesenchymal stem cells; hiPSC-SMCs: human induced pluripotent stem cell-derived smooth muscle cells; Tac: Tacrolimus; CsA: Cyclosporine; MMF: Mycophenolate mofetil; PSL: prednisolone; Methylene prednisolone: METH-PSL; NS: not specified.

Species; Gender	Sample Size	Model	Delivery	Cell Type	Immuno-Suppressive Therapy	Benefits	Adverse Events	Ref
Swine; NS	Sham *n* = 6 PBS *n* = 6 piPSC *n* = 6	MI	IM	piPSC	No	Cardiac autonomic nerve regeneration; less ventricular arrhythmia; myocardial perfusion; cardiac function.	No	[166,167]
Swine; NS	Sham *n* = 6 PBS *n* = 6 piPSC *n* = 6	MI	IM	piPSC	CsA + METH-PSL	Reduction of scar size; angiogenesis; less apoptosis and fibrosis.	No	[168]
Swine; NS	AGTP *n* = 6 Scaffold *n* = 13 Scaffold + AGTP *n* = 7 piPSC + AGTP *n* = 9 piPSC + Scaffold *n* = 11 piPSC + Scaffold + AGTP *n* = 11	MI	Scaffold + adipose pedicle (AGTP)	piPSC	No	None	No	[169]
Non-Human Primate; NS	MHC matched iPSC-CMs + TAC + MMF + PSL *n* = 2 MHC mismatched iPSC-CMs + TAC + MMF + PSL *n* = 3 MHC matched iPSC-CMs +TAC *n* = 1 MHC matched iPSC-CMs + No drug *n* = 1	Healthy	Sheet (back) vs. IM (heart)	iPSC-CMs	Tac + MMF + PSL	No host immune response in MHC-matched group + TAC + MMF + PSL.	No	[170]
Non-Human Primate; NS	PSC vehicle *n* = 5 MHC matched iPSC-CMs *n* = 5	MI	IM	iPSC-CMs	Tac+ METH-PSL	Cardiac function; less fibrosis; higher vascular density.	Ventricular arrhythmias	[114]
Non- Human Primate; NS	Sham *n* = 4 MHC matched iPSC-CMs *n* = 4 MHC mismatched iPSC-CMs *n* = 4	MI	Sheet	iPSC-CMs	Tac + MMF + PSL	Cardiac function; less fibrosis; higher vascular density.	No	[171]
Swine; NS	Sham *n* = 17 MI *n* = 17 Cells *n* = 17 Patch *n* = 17 Cells + Patch *n* = 17	MI	Sheet vs. IM	hiPSC-CMs hiPSC-ECs hiPSC-SMCs	CsA	Cardiac function; cardiac metabolism; higher vascular density; apoptosis reduction.	No	[172]
Swine; NS	Sham *n* = 5 Cells + Patch *n* = 5	MI	Sheet	hiPSC-CMs hiPSC-ECs hiPSC-SMCs	Yes; NS	Cardiac function; less fibrosis; higher vascular density.	No	[173]
Swine; NS	Sham *n* = 8 MI *n* = 15 Cells + Patch *n* = 13 Patch *n*= 14	MI	Sheet	hiPSC-CMs hiPSC-ECs hiPSC-SMCs	CsA + Methy-PSL	Cardiac function; less fibrosis; infarct size; less apoptosis.	No	[174]
Swine; Female	MI *n* = 9 hESC-CMs *n* = 10 hiPSC-MSCs *n* = 9	HF	IM	hiPSC-MSCs vs. hESC-CMs	CsA + steroid	Cardiac function; higher vascular density; less inflammation.	No	[175]
Swine; Female	Sham *n* = 6 Cell + Patch *n* = 6	MI	Sheet	hiPSC-CMs	Tac	Cardiac function; higher vascular density; less fibrosis.	No	[176]
Swine; Female	Cell + Patch *n* = 8 Cell + Patch + Omentum flap *n* = 8 Sham *n* = 5 Cell + Patch + Omentum flap *n* = 7 Cell + Patch *n* = 6 Omentum flap *n* = 5	MI	Sheet + omentum flap	hiPSC-MSCs hMSCs	Tac	Cardiac function; higher vascular density.	No	[177,178]
Swine; Female	MI *n* = 8; Tb4 *n* = 9; Tb4 Cell *n* = 8; Cell *n* = 8.	MI	Sheet + Tb4 microspheres	hiPSC-CMs	CsA	Cardiac function; higher vascular density; less fibrosis; reduction of scar size	No	[101]
Swine; Female	Sham *n* = 7 PBS *n* = 7 CCND2WTCMs *n* = 7 CCND2OECM *n* = 7	MI	IM	hiPSC-CCND2WTCMs vs. hiPSC-CCND2oeCMs	No	Host CM proliferation; angiogenesis in border zone; cardiac function; less fibrosis; reduced hypertrophy.	No	[179]
Swine; female	Healthy pigs *n* = 15 Healthy mini-pigs *n* = 20	Healthy	3D spheroids injection device	hiPSC-CMs	NS	Higher engraftment.	No	[180]
Swine; Female	Sham *n* = 5 Hydrogel *n* = 7 Cardiac spheroid *n* = 8	HF	3D spheroids injection device	hiPSC-CMs	Yes; NS	Cardiac function; infarct size reduction.	Ventricular arrhythmias	[103]
